# Hidden gems: a pilot project to solicit and reward patients’ and caregivers’ ideas for research

**DOI:** 10.1186/s40900-023-00473-y

**Published:** 2023-10-13

**Authors:** Freya Moxham, Christine Cutaran, Jakub Sadocha, Korey Capozza

**Affiliations:** 1 Global Parents for Eczema Research, Santa Barbara, USA; 2 International Alliance of Dermatology Patient Organizations, Ottawa, Canada

**Keywords:** Atopic dermatitis, Patient-centered research, AD, Research competition, Big ideas, Patient insights

## Abstract

**Background:**

Patients and caregivers investigate a wide range of approaches to address the signs and symptoms of their condition. Such investigation could lead to new treatment insights or avenues for research. However, currently there are few channels through which patients and families can share the results of their personal experiences; they need a platform to share their insights with the research community.

**Methodology:**

Two non-profit organizations, Global Parents for Eczema Research and the International Alliance of Dermatology Patient Organizations, developed a project to pioneer new ways for patients and caregivers to share their personal insights for research and for researchers and treatment developers to learn about those ideas. This project, the “Big Ideas for AD Research”, was a global challenge that awarded prizes and recognition to patients’ and caregivers’ research hypotheses related to atopic dermatitis management, treatment, and prevention.

**Results:**

The Challenge was open for 5 weeks and received 70 submissions from 11 countries. Entries were judged by two separate panels of experts that included both researchers and patients and caregivers. Winners were awarded with a monetary prize, recognized on social media, and connected by Global Parents for Eczema Research staff to an appropriate research group to help further their ideas.

**Conclusion:**

The Big Ideas for Eczema Challenge provided a proof of concept for a novel approach to bridging the gap between patients/caregivers and researchers/clinicians by developing a platform to garner the best ideas from the patient community for research. This model could be further leveraged by other patient groups to help solicit patients’ and caregivers’ ideas for research.

## Plain Language summary

Atopic Dermatitis (AD) is a chronic skin condition caused by inflammation that affects 1 in 5 children and 1 in 10 adults. Quality of life impacts, largely driven by itch and sleep loss, can be profound.

There are few channels through which patients and families of people with AD can share the results of their personal experiences and investigations; they need a platform to share their insights with the research community.

We outline a novel approach to engaging patients and their caregivers in research: a global research ideas challenge. This initiative, designed by two patient organizations, successfully identified high quality research ideas developed by patients and caregivers from their “lived experience”. Such ideas would not otherwise be apparent to the research community.

## Background

Atopic dermatitis (AD) is a chronic skin condition caused by inflammation that affects 1 in 5 children and 1 in 10 adults. Quality of life impacts, largely driven by itch and sleep loss, can be profound [1].

Patients and families impacted by atopic dermatitis (AD) often face challenges with finding acceptable treatments. These include limited treatment options; inadequate symptom resolution and/or concerning side effect profiles with existing treatments; and insufficient evidence to guide treatment and management decisions.

As a result, patients and caregivers often investigate a wide range of approaches to address the signs and symptoms of their condition [2–4].

Such investigation could lead to new treatment insights or avenues for research. However, currently there are few channels through which patients and families can share the results of their personal experiences and research with different treatment approaches.

These challenges, and the patient response, are not unique to the AD situation and are common among all disease areas where the research base is limited, few treatments are available, and patients lack adequate support to control symptoms.

Patients and caregivers need a platform to share their insights with the research community.

Our aim was to investigate the feasibility of developing and implementing a global research challenge competition designed for patients and parents.

## Methods

### Conception

Two non-profit organizations, global parents for AD research (GPER) and the International Alliance of Dermatology Patient Organizations, developed a project to pioneer new ways for patients and caregivers to share their personal insights for research and for researchers and treatment developers to learn about those ideas. This project, the “Big Ideas for AD Research”, was a global challenge that awarded prizes and recognition to patients’ and caregivers’ research hypotheses related to AD management, treatment, and prevention. It was developed by a 4-person project team consisting of patients and parents impacted by AD from two patient-focused organizations.

A scan of previous ‘Big Ideas’ competitions focused on other medical conditions and fields was carried out to see if any other organizations had previously conducted similar initiatives. None were identified that had a focus on both patients and caregivers and research applications. Nine related competitions were identified that had a similar focus, structure, or goals. Only three had a focus on research. Relevant parameters were reviewed and adapted from these three previous challenges: space exploration (National Institute of Aerospace, 2021), obstetrics (Saving Lives at Birth, 2022), and a multi-specialty research challenge (American Medical Association, 2022) [5–7].

### Developing contest guidelines

The project team considered and drafted the guidelines based on frameworks developed by other research ideas competitions and created a consent form with special attention to intellectual property, academic integrity, fairness, confidentiality, and privacy—adapting existing rules when available.

Entrants retained ownership of their ideas, but the competition was allowed to promote and share it via the host organizations’ communications as part of the Challenge.

These rules and guidelines were then reviewed and edited by an attorney who specialized in contest and sweepstakes law.

To help guide entrants with submitting appropriate ideas, submission categories were developed with explanations for what type of ideas would qualify for each. These included:

1. Mechanism of Disease (how AD “works”): This category was focused on ideas related to the underlying mechanisms of disease. Ideas could relate to biological and chemical pathways that could pinpoint causes and/or underlying mechanisms as to how and why AD occurs and/or why it subsides and relapses.
2. AD Treatment and Management: This included strategies and ideas for treating and managing AD.
3. AD Prevention: This category focused on strategies for preventing AD. This could include interventions to prevent AD at all or methods to prevent triggering events for AD.

Entrants had the opportunity to enter their idea to more than one category. Of a total of 70 entries 53 entered into just one category, with 40 in eczema treatment and management, 5 for Eczema prevention alone and 8 for Mechanism of disease alone. The remaining 17 were a combination of all three or two categories, with 15 for treatment and management, 14 for eczema prevention and 11 for mechanism of disease.

### Scoring criteria

The host organization staff then developed scoring criteria that both rewarded scientific merit and reflected patient-centred values such as ideas benefitting both patients and parents/caregivers and ingenuity that stemmed from patient and caregiver experience. The scoring criteria included: 1. Innovation/Novelty (how novel, innovative, or unique is the idea?) 2. Patient Centeredness (Does the idea stem from the patient and caregiver experience? Is the idea something that other patients/caregivers care about? Does it have the potential to benefit patients?) 3. Scientific Merit (Does the idea have a clear logic, rationale or hypothesis? Is the idea feasible and is there scientific basis for the idea?). 4. Clarity/Quality (Is the idea clearly stated and adequately developed with details and documentation).

Scoring criteria were included in the guidelines to guide entrants and were then used to help select the short list of ideas that would be reviewed by a panel of multi-disciplinary judges who scored each finalist idea.

### Building the platform

A separate competition website was developed that included the contest rules, consent form and submission platform. It was set up to disallow continuation until the consent form was completed. The platform and survey were developed in house and hosted on the GPER website and included questions asked were developed by GPER staff and a scientific advisory committee that is part of GPER. The submission platform consisted of a ten-question survey of multiple choice and short answer questions, these questions addressed the type of idea, how the entrant came up with their idea and their personal link to eczema. The competition was open for 32 days allowing patients and caregivers adequate time to develop and submit their ideas.

The competition was advertised on GPER and GlobalSkin’s social media channels, newsletter. Instagram posts from 2022 received 17,780 reviews across 8 GPER posts.

### Selection

The Challenge was open for 5 weeks and received 70 submissions from 12 countries. The host organization project team then removed all identifying information and assembled an internal review panel to screen the 70 submissions for initial selection.

The submissions were received from 12 countries with most to least entries being; USA(44), Canada(7), Australia(5), Serbia(5), Portugal(3), UK(3), Israel(1), Philippines(1), Belgium(1), Latvia(1), Sri Lanka(1), Kenya(1).

This first panel included 6 staff from the host organizations, five of whom were patients or parents and four who had science/research backgrounds. This panel eliminated incomplete entries and selected the top ideas based on the contest rules and scoring criteria; a review of the published literature to determine if each idea was “new” was also carried out. Submissions were ranked by the panel using the criteria included in the official rules and guidelines of the competition. The top 10 ideas were then sent to a judging panel for final review.

Briefly, the scoring criteria included categories relating to idea Novelty, Feasibility, Scientific Merit and Patient-Centredness.

### Judging

The judging panel consisted of an even split of patients/caregivers and researchers/clinicians to make sure patients had an equal voice in deciding the winning ideas. The judges were recruited from the GPER parent/patient and collaborator network and selected based on the pertinence of the professional backgrounds or education. In addition the panel included 4 physicians with dermatology training, 3 executive directors of eczema/dermatology groups and 6 parents/patients. Judges were from a wide range of geographical background including Canada, UK, USA, Portugal, Netherlands, Australia. Judges were offered an honorarium for their time, however, most of the judges did not accept the honorarium.

Both patients and caregivers along with researchers had equal weighting in the decision-making process. This kept patient perspectives as a priority whilst ensuring the ideas were of sufficient quality for further scientific research.

The strongest ideas were awarded with recognition, prize money, and help with connecting with research teams to advance their research idea.

Before the judging occurred, the team sent out a pre-meeting survey to the judges to further wean the top ideas from the initial selection list based on the same scoring criteria.

This resulted in 4 entries being chosen for in-depth discussion to ultimately choose the place winners for the competition. These ideas were discussed in a meeting with all the judges present in a virtual panel type format. During the one-hour panel judges advocated for, asked questions, and clarified the purpose and goals of each entry.

After much discussion and input from scientists and patients about what would be the most beneficial ideas to move forward with for the AD community and scientific progress, an anonymous poll took place where each judge got to vote with equal weighting. Each judge was able to vote for a first, second and 3rd place.

The finalist ideas can be found by accessing the Big Ideas website (ref) but briefly consisted of:

*First Prize*: Using isolated hookworm protein to trigger a re-balancing of the immune system from a parent of a child with eczema.

*Second Prize*: Preventing and counteracting exposure to pollutants that cause systemic oxidative damage and widespread inflammation in the skin by an adult with eczema.

*Third Prize*: Photobiomodulation and “red light therapy” for eczema by a parent of a child with eczema.

The winning ideas consisted of 3 people (2 men, 1 woman) all based in the USA, this is the consistent with a large majority of the entries being from the USA.

Winners and unsuccessful entrants were then contacted about the conclusion of the challenge. Winners were announced via a press release and promoted to the community via the Big ideas for Eczema Research website (www.bigideasforeczema.com) and on various social media platforms by both host organizations.

Global Parents for Eczema Research also produced a podcast in collaboration with the winners to further promote their ideas and release their idea to the scientific and caregiver community and share the inspiration and aspirations behind their proposals. This was released on the 9th of February 2023 and has received 501 downloads since publication to the time of writing.

Winners were awarded with a monetary prize of $4000 for 1st place, $2500 for 2nd place and $1000 for 3rd place, they were recognized on social media, and connected by Global Parents for Eczema Research staff to an appropriate research group to help further their ideas.

## Challenges

Challenges in developing this competition included communicating the opportunity and reaching patients and caregivers using social media channels. The idea that patients and caregivers can contribute to science is likely new to many people and requires explanation that isn’t always easy to do through short social media posts. However, by breaking up the messages over several posts we were able to set the stage and communicate the rationale.

In addition, staff time was required to vet entries and screen out those that were submitted by commercial interests or non-parents/patients.

Both a concern from the project team and winners was the challenge of moving winning ideas from concept to actual research collaborations given that funding wasn’t attached to the award for research and winners may be new to the research process.

To combat this, when the competition was repeated in 2023, we sought the opportunity for winners to partake in the patient led opportunity training (PLOT) programme empowering parents and patients in developing their skills as citizen scientists which will show them how to engage with research groups and apply for bigger research grants to further their proposals. The PLOT programme is provided by Stanford medical school with funding from Pfizer and is being offered to 2022 and 2023 winners and developed by Stanford in collaboration with the GPER project staff.

## Patient and public involvement

Patient and caregiver involvement was built into the project because it was conceived of and developed by a team that consisted entirely of this group, and the selected process was driven by a panel of judges where patients and caregivers comprised 50% of the panel.

## Conclusions

After collecting 70 submissions from 11 different countries over 5 weeks, the Big Ideas for Eczema Challenged identified novel and promising ideas derived from the patient experience. The parent and caregiver “citizen scientists” were paired with research groups within the selected field of their idea, and new research collaborations may come out of the Challenge.

There was no pre-determined success criteria for the project due to its novelty however the project was considered a success as it both helped patients' voices be heard and bought novel ideas to the research community allowing for progression in the field whilst prioritising patient views and ideas. This project has bought new ideas to the forefront ideas that wouldn’t otherwise be captured or shared with the research community.

This competition has helped to bridge the gap between patients/caregivers and researchers/clinicians by finding a middle ground and starting relationships that aim to benefit both the patient and researcher communities. This model could be further leveraged by other patient groups to help solicit patients’ and caregivers’ ideas for research.

## Data Availability

Available upon request.
